# Amifostine reduces inflammation and protects against 5-fluorouracil-induced oral mucositis and hyposalivation

**DOI:** 10.1590/1414-431X20188251

**Published:** 2019-02-25

**Authors:** S.C.M. Barbosa, V.B.M. Pereira, D.V.T. Wong, A.P.M. Santana, L.T. Lucetti, L.L. Carvalho, C.R.N. Barbosa, R.B. Callado, C.A.A. Silva, C.D.H. Lopes, G.A.C. Brito, N.M.N. Alencar, R.C.P. Lima-Júnior

**Affiliations:** 1 Departamento de Fisiologia e Famacologia, Faculdade de Medicina, Universidade Federal do Ceará, Fortaleza, Brasil; 2 Departamento de Patologia e Medicina Legal, Faculdade de Medicina, Universidade Federal do Ceará, Fortaleza, Brasil; 3 Departamento de Morfologia, Faculdade de Medicina, Universidade Federal do Ceará, Fortaleza, Brasil

**Keywords:** Cancer chemotherapy toxicity, Oral mucositis, 5-Fluorouracil, Amifostine, Cytokines

## Abstract

Oral mucositis (OM) is a common and dose-limiting side effect of cancer treatment, including 5-fluorouracil (5-FU) and radiotherapy. The efficacy of the therapeutic measures to prevent OM is limited and disease prevention is not fully observable. Amifostine is a cytoprotective agent with a described anti-inflammatory potential. It is clinically used to reduce radiotherapy and chemotherapy-associated xerostomia. This study investigated the protective effect of amifostine on an experimental model of OM. Hamsters were divided into six groups: saline control group (5 mL/kg), mechanical trauma (scratches) of the right cheek pouch; 5-FU (60 and 40 mg/kg, *ip*, respectively, administered on days 1 and 2); amifostine (12.5, 25, or 50 mg/kg) + 5-FU + scratches. Salivation rate was assessed and the animals were euthanized on day 10 for the analysis of macroscopic and microscopic injury by scores. Tissue samples were harvested for the measurement of neutrophil infiltration and detection of inflammatory markers by ELISA and immunohistochemistry. 5-FU induced pronounced hyposalivation, which was prevented by amifostine (P<0.05). In addition, 5-FU injection caused pronounced tissue injury accompanied by increased neutrophil accumulation, tumor necrosis factor-alpha (TNF-α), and interleukin-1 beta (IL-1β) tissue levels, and positive immunostaining for TNF-α, IL-1β, and inducible nitric oxide synthase (iNOS). Interestingly, amifostine prevented the inflammatory reaction and consequently improved macroscopic and microscopic damage (P<0.05 *vs* 5-FU group). Amifostine reduced inflammation and protected against 5-FU-associated oral mucositis and hyposalivation.

## Introduction

Oral mucositis (OM) is a side effect related to radiation and cancer chemotherapy. Patients experience pain, xerostomia, erythema, and ulceration of oral mucosa, which, together with leucopenia, increase the risk of bacteremia and sepsis (1,2). OM incidence and severity depends on the anticancer drug used, the existence of poor oral hygiene, and the presence of chronic periodontal disease at the time of treatment initiation (3).

5-Fluorouracil (5-FU) is an antimetabolite drug used for tumors of the breast, head and neck, and digestive tract administered in regimens that include irinotecan or oxaliplatin (4). Patient tolerability to 5-FU administration is commonly reduced due to myelosuppression, and oral and intestinal mucositis (5). In general, OM affects more than 40% of patients undergoing 5-FU-based therapy (6), limiting dose-intensity and reducing patient quality of life (7).

The pathogenesis of OM includes the direct damage to the mucosa induced by oxidative stress and the activation of transcription factors, such as nuclear factor-kappa B (NF-κB), which increases the expression of proinflammatory mediators, including tumor necrosis factor-alpha (TNF-α), interleukin-1 beta (IL-1β), interleukin-6 (IL-6), cyclooxygenase-2 (COX-2), and inducible nitric oxide synthase (iNOS) (8–10). The production of inflammatory markers along with a pronounced inflammatory infiltrate culminate with mucosal ulceration and barrier disruption (11). Clinical measures currently used to treat OM include oral cryotherapy, keratinocyte growth factor-1, low-level laser therapy, and analgesics (12). However, the efficacy of these therapeutic approaches is limited and disease prevention is not fully observable.

Amifostine, also known as WR-2721, is a prodrug that is dephosphorylated by alkaline phosphatase and acts as a broad-spectrum cytoprotective agent by scavenging free radicals, protecting cell membranes, and preventing DNA damage. In the clinical setting, amifostine is employed to reduce radiotherapy and chemotherapy-associated xerostomia (13,14). This drug was reported to prevent inflammation in experimental models of anticancer-related side effects, such as hemorrhagic cystitis (15), and also to reduce neutrophil accumulation in gastric lesions induced by indomethacin (16). Despite the suggestion that amifostine could prevent oral mucositis in patients with head and neck cancer receiving treatment, conclusions are conflicting (17).

In a systematic review by Gu and co-workers, amifostine reduces severe mucositis and xerostomia (18). Currently, most of the studies have assessed the protection of amifostine against OM induced by chemotherapy (paclitaxel/carboplatin) and radiotherapy (19), but no evidence of protection on 5-FU-related mucositis and xerostomia has been demonstrated. In addition, the pathogenesis of chemotherapy-associated side effects differs according to the drug employed (20). Therefore, this study aimed to investigate the protective effect of amifostine on 5-FU-induced OM and the possible underlying mechanisms of protection.

## Materials and Methods

### Animals and ethics statement

Male Golden Syrian hamsters (100–150 g) from the animal facility of the Federal University of Ceará were kept in appropriate cages in temperature controlled rooms with 12-h light-dark cycles and received food and water *ad libitum*. Experimental procedures complied with the laboratory animal care and the principles outlined by the National Institutes of Health and were approved by the ethics committee for Animal Experiments (protocol number 01/2011) of the Universidade Federal do Ceará.

### Induction of experimental oral mucositis

Oral mucositis was induced by two intraperitoneal (*ip*) injections on the first and second experimental days, using 60 and 40 mg/kg of 5-FU, respectively. This protocol was based on a model previously described by Sonis and co-workers (21) and modified for our experimental conditions (8,9). In order to mimic the friction to which oral mucosa is normally subjected in a daily routine, the right cheek pouch of the animals were superficially scratched with the tip of a 22-gauge needle on the fourth day. This procedure was conducted under anesthesia with 2.5% tribromoethanol solution (10 ml/kg, *ip*). The needle was dragged twice in a linear movement across the everted cheek pouch until erythematous changes were noted.

**Figure 1:**
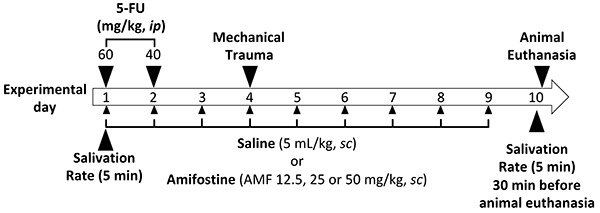
Schematic diagram of the experimental protocol.

Hamsters were randomly assigned into six groups (n=6/group): Control group: injected with 0.9 % saline (5 mL/kg, *sc*, daily for 9 days); Mechanical trauma: animals subjected to scratches (mechanical trauma of the right cheek pouch) on day 4 and treated with 0.9% saline (5 mL/kg, *sc* , from day 1 to 9); 5-FU: intraperitoneal injection of 5-FU (60 and 40 mg/kg, respectively, on days 1 and 2), subjected to scratches (mechanical trauma of the right cheek pouch) on day 4, and treated with 0.9% saline (5 mL/kg, *sc*, from day 1 to 9); amifostine (AMF): intraperitoneal injection of 5-FU (60 and 40 mg/kg, respectively, on days 1 and 2), subjected to scratches (mechanical trauma of the right cheek pouch) on day 4, and treated with 12.5, 25, or 25 mg/kg AMF, *sc*, from day 1 to 9. On days 1 and 2, AMF was injected 30 min before 5-FU. The animals were euthanized on day 10 and samples of the cheek pouch were excised for macroscopic analysis, histopathology, immunohistochemistry, and biochemical assays. Figure 1 depicts a schematic diagram of the experimental protocol.

### Hyposalivation assay

The hamsters were anesthetized with 2.5% tribromoethanol (10 mL/kg, *ip*) on days 4 and 10 for evaluation of the salivation rate (22). The pre-existing saliva in the oral cavity was removed with cotton balls positioned in the mouth floor. Then, filter paper cones (Tanari Industrial LTDA, Brazil) for endodontic use were weighed (time 0 min) and placed bilaterally in the mouth floor for 5 min for the absorption of secreted saliva. Thereafter, the cones were removed and weighed again (time 5 min). The saliva production was determined by the difference between the two measurements (time 5 min – time 0 min). The variation on the salivation rate was determined by the difference between measurements at days 0 and 10 and expressed in milligram.

### Macro- and microscopic analysis of cheek pouch

The cheek pouches were everted and photographed for macroscopic analysis. Signs of injury, such as erythema, hyperemia, hemorrhagic areas, epithelial ulcerations, and the presence of abscess were scored by a person (DVTW) who was unaware of the treatments, as follows: Score 0: healthy mucosa and no evidence of erosion or vasodilation; Score 1: presence of erythema and no evidence of mucosal erosion; Score 2: severe erythema, vasodilatation, and superficial erosion; Score 3: presence of ulcers in one or more surfaces of the mucosa, affecting no more than 25% of the area, severe erythema, and vasodilatation; Score 4: ulcers in about 50% of the area of the jugal mucosa; Score 5, completely ulcerated jugal mucosa that made it impossible to expose the tissue (23).

Then, the animals were euthanized by an injection of xylazine (10 mg/kg, *ip*) and ketamine (80 mg/kg, *ip*) followed by exsanguination through the abdominal aorta. Cheek pouch samples were collected for histopathological analysis. The specimens were fixed in 10% (v/v) neutral-buffered formalin, dehydrated, and embedded in paraffin. Sections (5-μm thick) were obtained for hematoxylin-eosin staining (H&E) and examined under light microscopy (40× and 100×). Inflammatory cell infiltration, vasodilatation, presence of hemorrhagic areas, edema, ulcerations, and abscesses were blindly analyzed by a pathologist (GACB) who was unaware of the treatments, as follows: Score 0: normal epithelium and connective tissue without vasodilatation, absence of or mild cellular infiltration, absence of hemorrhagic areas, ulcerations, or abscesses; Score 1: mild vasodilatation, reepithelization areas, mild inflammatory infiltration with prevalence of mononuclear cells, absence of hemorrhagic areas, edema, ulcerations or abscesses; Score 2: moderate vasodilatation, areas of hydropic epithelial degeneration, inflammatory infiltration with neutrophil prevalence, presence of hemorrhagic areas, edema, and eventual ulcerations, absence of abscesses; Score 3: severe vasodilatation, inflammatory infiltration with neutrophil prevalence, presence of hemorrhagic areas, edema, and extensive ulceration, and abscesses (8).

### Myeloperoxidase assay

Myeloperoxidase (MPO) was used as an inflammatory infiltration marker. Briefly, samples were homogenized in HTAB buffer (Sigma-Aldrich, Brazil) and centrifuged at 2500 *g* for 7 min at 4°C. The pellet was resuspended and the MPO activity was assayed by measuring the change in absorbance at 450 nm using an o-dianisidine dihydrochloride and 1% H_2_O_2_ mixture (t_0_=0 min and t_1_=1 min). The absorbance change was recorded, plotted on standard MPO curve, and the values obtained are reported as MPO activity U/mg of tissue. A unit of MPO was defined as the amount of enzyme required to convert 1 µmol/min of H_2_O_2_ into water at 22°C (24).

### Detection of tumor necrosis factor-alpha (TNF-α) and interleukin-1 beta (IL-1β) by ELISA

Mucosal samples were homogenized and processed as described by Melo and colleagues (25). The microtiter plates were coated at 4°C overnight with a mouse anti-rat TNF-α or a goat anti-rat IL-1β (0.8 μg/mL) primary antibody (R&D System, USA). After incubation with the blocking solution 5% BSA (bovine serum albumin), the samples and standards were added in duplicate and incubated at 4°C for 2 h. The plates were washed three times with buffer and the biotinylated detection antibody (diluted 1:1000 with 1% BSA assay buffer, R&D System). After incubation at room temperature for 2 h, the plates were washed and 100 μL of streptavidin-HRP (1:200 dilution) and 100 µL of substrate solution (1:1 mixture of H_2_O_2_ and tetramethylbenzidine; R&D System) were added to the plate, which was incubated in a dark at room temperature for 20 min. The reaction was stopped with 2N H_2_SO_4_ and the absorbance was measured at 450 nm. The results are reported as pg/mg of tissue.

### Immunohistochemistry for TNF-α, IL-1β, and inducible nitric oxide synthase (iNOS)

Immunohistochemistry was performed using the streptavidin-biotin-peroxidase as described previously (26). Samples were fixed in 10% formalin for 24 h. They were then dehydrated, embedded in paraffin, and sectioned. The sections were deparaffinized and rehydrated in xylene and alcohol. After antigen retrieval and blockade of endogenous peroxidase, the slides were incubated overnight at 4°C with primary goat anti-TNF-α, rabbit anti-IL1-β, or rabbit anti-iNOS antibodies (Santa Cruz Biotechnology, USA) diluted 1:100 in 5% BSA. Then, the slides were washed and incubated for 30 min with biotinylated rabbit anti-goat (TNF-α) or goat anti-rabbit (IL-1β or iNOS) secondary antibodies (1:400 dilution) (Santa Cruz Biotechnology). TNF-α, IL-1β, and iNOS staining was visualized with chromogen 3,3′-diaminobenzidine (Dako, Agilent Technologies, USA). The slides were counterstained with Harry's hematoxylin, dehydrated in a graded alcohol series, cleared in xylene and cover-slipped. Immunostained cells were scored as follows: 0) absence of labeled cells; *1*) weak staining; *2*) moderate, or *3*) strong immunostaining.

### Statistical analysis

Statistical analysis was performed using GraphPad Prism^®^ software, version 6.0 (USA). The data were submitted to analysis of variance (ANOVA) followed by Newman-Keuls' test (parametric data) or to Kruskal-Wallis followed by Dunn's test (non-parametric data). The results are reported as means±SEM (parametric data) or as median (minimum-maximum) (non-parametric data). Differences between groups were considered statistically significant at P<0.05.

## Results

### Amifostine restored normal salivation rate

**Figure 2:**
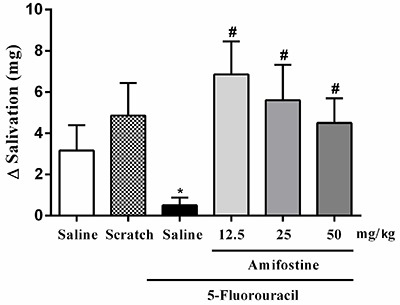
Amifostine improved 5-fluorouracil (5-FU)-associated hyposalivation. Data are reported as means±SE (n=6/group). *P<0.0*5 vs* saline group or scratch. ^#^P<0.05 *vs* 5-FU-treated group.

Figure 2 shows that 5-FU significantly (P<0.05) reduced the salivary rate (0.50±0.38) compared with the saline group (3.17±1.22). In addition, AMF pre-treatment significantly prevented such reduction at all doses tested (P<0.05) compared to the 5-FU-injected group (12.5 mg/kg: 226%; 25 mg/kg: 173; 50 mg/kg: 133%). Mucosal scratching did not change the salivation rate *per se* compared with the saline control group (P>0.05)

### Amifostine improved 5-FU-associated macroscopic and microscopic injury

**Figure 3:**
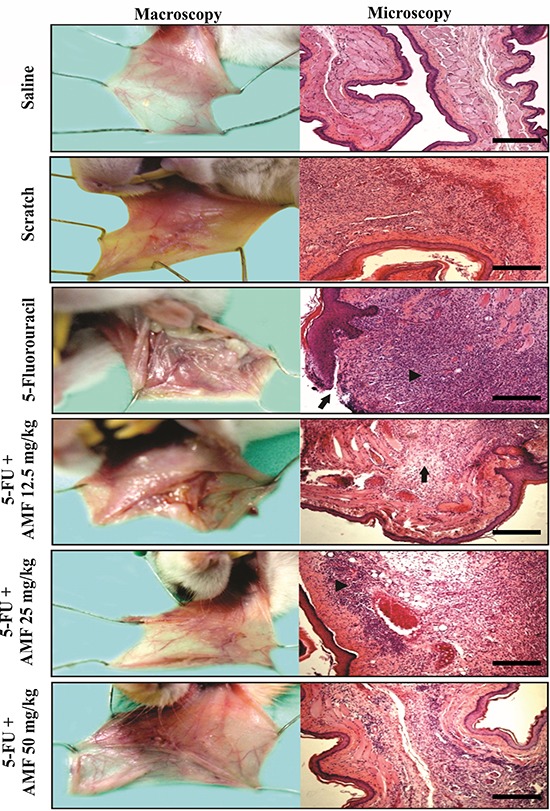
5-fluorouracil (5-FU) induced macroscopic and microscopic injury, which was prevented by amifostine. The saline-treated and scratch groups showed no sign of tissue damage. Conversely, 5-FU significantly induced tissue erythema, mucosal ulceration, abscesses, and hemorrhage versus the saline group. In addition, amifostine (AMF) protected the animals from the macroscopic and microscopic damage compared to the 5-FU group. Arrowheads indicate areas of inflammatory infiltration. Arrows denote edema and ulceration areas. Magnification: 40×, scale bar: 500 μm.

As depicted in Figure 3 and Table 1, 5-FU significantly induced tissue erythema, mucosal ulceration affecting more than 25–50% of the mucosa, presence of abscesses and hemorrhage as detected by macroscopy (5[3–5]) and histopathology (3[2–3]) compared with the saline group (macroscopy: 0[0-0] and histopathology: 0[0–0], P<0.05). The group that was submitted only to scratch showed no significant sign of tissue damage compared to the saline group (P>0.05). Conversely, AMF significantly protected the animals from macroscopic and microscopic injury compared to the 5-FU group (P<0.05).

**Table 1:** Amifostine (AMF) prevented 5-fluorouracil (5-FU)-associated macroscopic and microscopic injury.

Groups	Macroscopic scores	Microscopic scores
Saline	0 (0–0)	0 (0–0)
Scratch	1 (1–3)	2 (0–3)
5-FU + Saline	5 (3–5)*	3 (2–3)*
5-FU + AMF 12.5 mg/kg	3.5 (1–5)	2 (0–3)^#^
5-FU + AMF 25 mg/kg	2 (1–4)^#^	2.5 (1–3)

Data are reported as median and range of at least six samples. *P<0.05 *vs* saline group. ^#^P<0.05 *vs* 5-FU group. Data were analyzed by Kruskal-Wallis and Dunn's tests.

### Amifostine attenuated neutrophil infiltration and the levels of inflammatory markers

**Figure 4:**
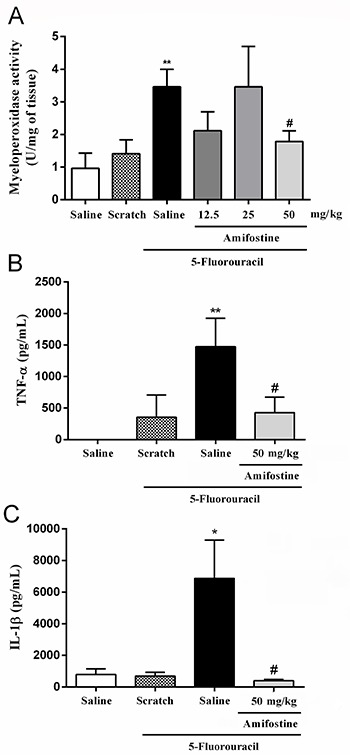
5-fluorouracil (5-FU)-induced inflammation was abolished in amifostine-treated animals. The proinflammatory effect of 5-FU upon the cheek pouch was detected as an increase in myeloperoxidase activity (**A**) and the production of tumor necrosis factor-alpha (TNF-α) (**B**) and interleukin-1 beta (IL-1β) (**C**). Data are reported as means±SE (n=6/group). *P<0.05, **P<0.01 *vs* group treated only with saline or scratch. ^#^P<0.05 *vs* 5-FU-treated group.

5-FU significantly increased (P<0.05) the MPO activity, indicating a pronounced neutrophil accumulation, and also augmented tissue levels of TNF-α and IL-1β (MPO: 3.5±0.5 U/mg of tissue; TNF-α: 1471±752 pg/mg of tissue; IL-1β: 6870±2417 pg/mg of tissue) *vs* the saline group (MPO: 0.9±0.5; TNF-α: 0.0±0.0 pg/mg of tissue; IL-1β: 787.5±356.1 pg/mg of tissue). Additionally, daily injection of AMF (50 mg/kg) attenuated (P<0.05) MPO activity (64%, Figure 4A) and reduced tissue levels of TNF-α (70% reduction Figure 4B) and IL-1β (93% reduction, Figure 4C) compared to the 5-FU group (Figure 4A-C). Remarkably, the group submitted to control mechanical trauma showed no sign of inflammation (MPO: 1.4±0.4 U/mg of tissue; TNF-α: 354.3±354.3 pg/mg of tissue; IL-1β: 691.3± 239.5 pg/mg of tissue) compared with the saline group (P>0.05, Figure 4A-C), indicating that the scratch was not enough to cause oral mucositis.

### Amifostine reduced TNF-α, IL-1β, and iNOS immunostaining in the cheek pouch

**Figure 5:**
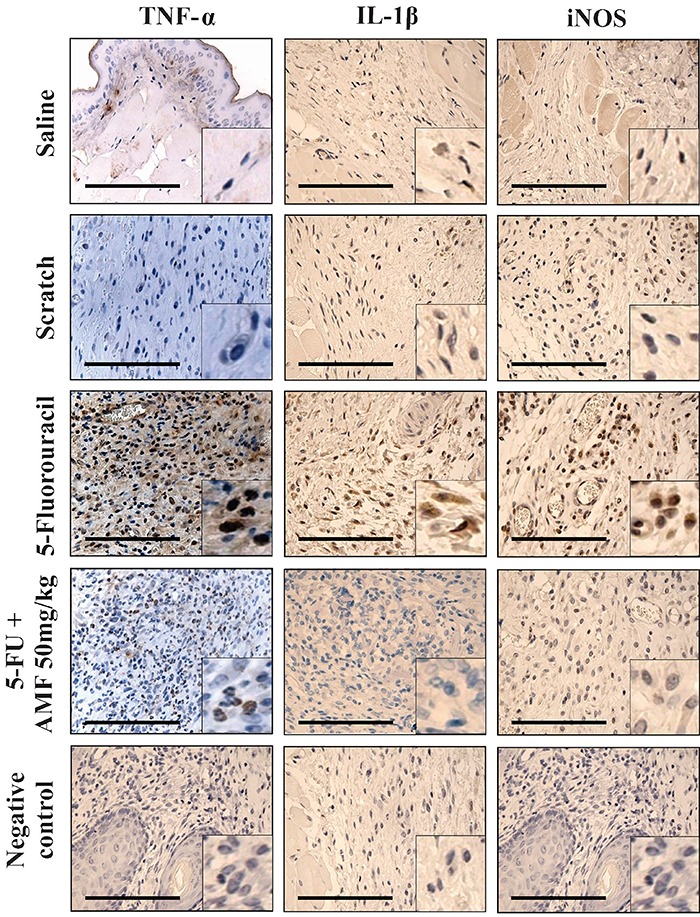
Amifostine (AMF) reduced tumor necrosis factor-alpha (TNF-α), interleukin-1 beta (IL-1β), and inducible nitric oxide synthase (iNOS) immunostaining. Magnification: 100×, scale bar: 250 μm. Representative stained cells are depicted in the lower right side of each panel (magnification 400×).

Figure 5 shows representative immunostaining photomicrographs of inflammatory markers and Table 2 summarizes the results of a semi-quantitative analysis of the immunoexpression for these inflammatory mediators. Consistently, 5-FU increased the intensity of TNF-α, IL-1β, and iNOS stained cells [2(1–3); 2(1–3); 3(2–3), respectively], compared to the saline group [TNF-α: 0(0–0); IL-1β: 0(0–1); iNOS: 0(0–1)] (P<0.05). In addition, most of the cells were unstained or weakly stained in the AMF-treated group, which differed from the 5-FU group (P<0.05).

**Table 2:** Immunostaining scores for tumor necrosis factor-alpha (TNF-α), interleukin-1 beta (IL-1β), and inducible nitric oxide synthase (iNOS).

Groups	Scores		
	TNF-α	IL-1	iNOS
Saline	0 (0–0)	0 (0–1)	0 (0–1)
Scratch	0 (0–1)	1 (1–2)	2 (0–3)
5-FU+ Saline	2 (1–3)*	2 (1–3)*	3 (2–3)*
AMF 50 mg/kg + 5-FU	0 (0–1)^#^	0 (0–2)^#^	1 (0–3)^#^

Data are reported as median and range of six samples. *P<0.05 *vs* saline group. ^#^P<0.05 *vs* 5-FU group. Data were analyzed by Kruskal-Wallis and Dunn's tests. 5-FU: 5-fluorouracil; AMF: amifostine.

## Discussion

In this study, 5-FU induced oral mucositis, leading to a pronounced inflammatory response and reduction of salivary rate. Interestingly, such parameters of tissue injury were prevented by amifostine treatment.

First, an experimental model of mucositis in hamsters was used to evaluate the cytoprotective effect of amifostine (9). This animal model is suitable for the study of chemotherapy-associated oral mucositis pathogenesis, since the structure of the hamster's cheek mucosa is separated from the pouch and also presents loose connective tissue. These characteristics allow the pouch to be turned outward for induction of tissue injury and follow-up of damage (27). Consistently, the animals that received 5-FU followed by mechanical trauma of the cheek presented macroscopic and microscopic lesions accompanied by an inflammatory response with accumulation of TNF-α, IL-1β, iNOS, and reduced salivary rate, indicating the establishment of ongoing oral mucositis.

Current guidelines recommend the use of amifostine for the clinical management of radiation proctitis and esophagitis induced by concomitant chemotherapy and radiation therapy (28). Conversely, there is no specific recommendation for the use of amifostine to prevent oral mucositis due to the conflicting evidence (12,19,29). Considering the lack of literature evidence to support the use of amifostine for 5-FU-related oral mucositis management, that drug was investigated in an experimental model of mucositis. Interestingly, amifostine prevented both inflammation and reduced salivary rate. Such a positive response was previously reported in a randomized clinical study in which advanced head and neck cancer patients received radiochemotherapy and were analyzed for oral cavity toxicities (30). Consistently, amifostine-treated individuals showed a lower incidence of severe mucositis and xerostomia compared with non-treated patients (30). However, those previous reports failed to investigate the underlying mechanisms through which amifostine prevents mucositis.

Several studies reported that amifostine prevents inflammation in different animal models, such as acrolein- or ifosfamide-induced hemorrhagic cystitis (15) and indomethacin- or ethanol-associated gastric lesions (16,31). The protective mechanism of amifostine is accompanied by the reduction of neutrophil accumulation in target organs (15,16). Consistently, amifostine prevented neutrophil infiltration in the oral mucosa, which was associated with reduced microscopic and macroscopic injury. Considering that only the highest dose of amifostine prevented neutrophil oral mucositis, that dose was used for subsequent inflammatory assays.

Cytokines are known inflammatory markers that participate in the pathogenesis of 5-FU-induced oral mucositis (8,32). Lima and co-workers suggested the involvement of TNF-α by the use of thalidomide, a drug that enhances the degradation of TNF-α messenger RNA (8). The role of other cytokines, such IL-β, IL-6, and IL-8, was also confirmed when the animals were treated with pentoxyphylline, a pan-inhibitor of cytokines (8). The expression of these cytokines is mediated by the activation of NF-κB, which amplifies the mechanisms of tissue damage (32). Consistently, dexamethasone, a potent glucocorticoid that inhibits NF-κB signaling, was also shown to prevent 5-FU-induced oral mucositis, indicating the strong inflammatory component of such disease (10). Interestingly, amifostine prevented the immunoexpression of key inflammatory markers, such as TNF-α, IL-1β, and iNOS, confirming the anti-inflammatory effect of that drug and suggesting the potential applicability to manage oral mucositis.

In summary, we showed that in addition to managing hyposalivation, amifostine prevented oral mucositis by reducing the immunoexpression of inflammatory mediators with a well-known role in the pathogenesis of that side effect. Considering the reduced effectiveness of clinical measures to treat mucositis, amifostine could be an important alternative approach for the management of this condition.
